# A Novel Deep Learning-Based (3D U-Net Model) Automated Pulmonary Nodule Detection Tool for CT Imaging

**DOI:** 10.3390/curroncol32020095

**Published:** 2025-02-08

**Authors:** Abhishek Mahajan, Rajat Agarwal, Ujjwal Agarwal, Renuka M. Ashtekar, Bharadwaj Komaravolu, Apparao Madiraju, Richa Vaish, Vivek Pawar, Vivek Punia, Vijay Maruti Patil, Vanita Noronha, Amit Joshi, Nandini Menon, Kumar Prabhash, Pankaj Chaturvedi, Swapnil Rane, Priya Banwar, Sudeep Gupta

**Affiliations:** 1 Department of Imaging, The Clatterbridge Cancer Centre NHS Foundation Trust, Liverpool L7 8YA, UK; 2 Faculty of Health and Life Sciences, University of Liverpool, Liverpool L69 3BX, UK; 3 Department of Radiodiagnosis and Imaging, Tata Memorial Hospital, Homi Bhabha National Institute, Mumbai 400012, India; drrajattmh2019@gmail.com (R.A.); ujjwalagg8@gmail.com (U.A.); renukaashtekar1@gmail.com (R.M.A.); priyabanwar@gmail.com (P.B.); 4 Endimension Technology Pvt Ltd., Maharashtra 400076, India; bharadwaj_kss@endimension.com (B.K.); apparao_mlv@endimension.com (A.M.); vivek_pawar@endimension.com (V.P.); vivekpoonia@endimension.com (V.P.); 5 Department of Surgical Oncology, Tata Memorial Hospital, Mumbai 400012, India; drmahajanricha@gmail.com (R.V.); chaturvedi.pankaj@gmail.com (P.C.); 6 Department of Medical Oncology, Tata Memorial Hospital, Mumbai 400012, India; vijaypgi@gmail.com (V.M.P.); vanita.noronha@gmail.com (V.N.); amitjoshi74@gmail.com (A.J.); nandini.menon1412@gmail.com (N.M.); kprabhash1@gmail.com (K.P.); sudeepgupta04@yahoo.com (S.G.); 7 Department of Pathology, Tata Memorial Hospital, Mumbai 400012, India; raneswapnil82@gmail.com

**Keywords:** pulmonary nodule, deep learning, radiology, assistive technology, U-Net, deep convolutional neural networks

## Abstract

Background: Precise detection and characterization of pulmonary nodules on computed tomography (CT) is crucial for early diagnosis and management. Objectives: In this study, we propose the use of a deep learning-based algorithm to automatically detect pulmonary nodules in computed tomography (CT) scans. We evaluated the performance of the algorithm against the interpretation of radiologists to analyze the effectiveness of the algorithm. Materials and Methods: The study was conducted in collaboration with a tertiary cancer center. We used a collection of public (LUNA) and private (tertiary cancer center) datasets to train our deep learning models. The sensitivity, the number of false positives per scan, and the FROC curve along with the CPM score were used to assess the performance of the deep learning algorithm by comparing the deep learning algorithm and the radiology predictions. Results: We evaluated 491 scans consisting of 5669 pulmonary nodules annotated by a radiologist from our hospital; our algorithm showed a sensitivity of 90% and with only 0.3 false positives per scan with a CPM score of 0.85. Apart from the nodule-wise performance, we also assessed the algorithm for the detection of patients containing true nodules where it achieved a sensitivity of 0.95 and specificity of 1.0 over 491 scans in the test cohort. Conclusions: Our multi-institutional validated deep learning-based algorithm can aid radiologists in confirming the detection of pulmonary nodules through computed tomography (CT) scans and identifying further abnormalities and can be used as an assistive tool. This will be helpful in national lung screening programs guiding early diagnosis and appropriate management.

## 1. Introduction

Lung cancer is one of the most frequent cancers in the world, being the most commonly diagnosed cancer and the leading cause of cancer-related mortality. It is the most regularly diagnosed cancer in the male population in India and the fifth most usually diagnosed cancer in the female population [1,2]. The outcome of lung cancer remains the poorest among all the 5-year survival rates of about 10–20%, the rates being largely dependent on the stage of detection ranging from 0 to 97% from later stages to the earliest ones. The proposed reason for its low survival is its aggressive nature and detection in advanced stages [3]. Therefore, early detection gives an important chance to improve overall survival; however, its use has had limited success with less sensitivity. Many studies have used low-dose MDCT and shown significant benefits compared to the rest of the available methods like chest radiography and sputum cytology [4,5]. The detection and characterization of the pulmonary nodules are well established and performed by trained authorized radiologists in a clinical setup. However, in the recent ongoing trend of mass lung cancer screening trials or in a setup with a high patient load, the already limited number of trained radiologists can be a limiting factor in detecting these pulmonary lesions among hundreds of scans. This can lead to an increase in the rates of missed findings due to multiple reasons including nodule characteristics, image quality, or perception error by the radiologist, which could be due to scanning, recognition, or decision-making errors in addition to reasons like reading conditions, fatigue, or distraction [6,7,8]. This highlights the need for technological advancements to reduce workload and improve nodule screening sensitivity while minimizing false positives [9]. Deep learning-based computer-aided detection (CAD) systems have been developed and tested to assist radiologists by automatically detecting and locating pulmonary nodules, offering promising AI-driven methods for image analysis [10]. By exploring these new pathways, radiologists are likely to play a leading role in medical applications of AI [11]. AI encompasses complex processes like hypothesis generation, data analysis, testing, and program creation through machine learning (ML), neural networks, and deep learning. ML, a subset of AI, enables computers to learn from data without explicit programming and is widely used in medical imaging.

Deep learning algorithms are new in AI research and do not require explicit feature definition, representing a fundamentally different paradigm in machine learning [12]. With ample data and computational power, deep learning algorithms have advanced significantly, learning directly from data without predefined features. Among various architectures, convolutional neural networks (CNNs) are the most widely used in medical imaging [13].

Rafael Wiemker from Philips Research highlighted CAD’s potential in early cancer detection and nodule diagnosis based on morphology and volume changes, noting that improved spatial and temporal CT resolution enhances algorithm performance in pulmonary nodule detection [14]. Conventional CAD systems showed low sensitivity and high false positives, prompting a shift to advanced deep learning models. These systems provide secondary predictions to aid final decision-making [15,16,17]. Our study aims at developing such an algorithm using a 3D deep convolutional neural network and further testing to validate its performance in a given dataset to increase its diagnostic performance in routine practice.

## 2. Materials and Methods

The study was conducted in collaboration with a tertiary cancer center and the study was approved by the Institutional Ethics Committee. The need for obtaining informed consent was waived, and data collection and storage were performed in accordance with the local guidelines. Data were anonymized to keep the personal information of patients confidential. The study was performed in accordance with the ethical guidelines outlined in the Declaration of Helsinki, Good Clinical Practice guidelines, and the Indian Council of Medical Research guidelines. The study is not registered in the clinical trial registry. No funding was used for the purpose of this study.

### 2.1. Variables

To measure the performance of our deep learning model, we used measures like specificity, sensitivity, FROC curve, and CPM score.

### 2.2. Definitions

Specificity: Fraction of positive ground truths covered by the predictions.

Sensitivity: Fraction of negative ground truths covered by the predictions.

FROC curve: Free-response receiver operating characteristic (FROC) analysis [18]. In the FROC curve, sensitivity is plotted as a function of the average number of false positives per scan (FPs/scan).

CPM score: The competition performance metric (CPM) is defined as the average sensitivity at seven predefined FPs/scan rates: 1/8, 1/4, 1/2, 1,2,4, 8.

### 2.3. Study Methodology

We used the standard procedure for evaluating deep learning-based model systems in this study. We used a dataset provided by our institute and a publicly available dataset. The entire AI pipeline consists of different stages and training models with different types of loss functions.

Our entire AI pipeline consists of two stages of processing the computed tomography (CT) scans. The first stage predicts and segments the pulmonary nodules using a deep convolutional neural network (DCNN). The DCNN architecture with which we decided to segment the pulmonary nodules is a variant of U-Net [17], a state-of-the-art deep convolutional network. The first stage is referred to as the detection stage. The second stage uses a similar U-Net-like deep convolutional network as the first stage, but it is coupled with a classification output to determine whether the nodule identified by the first stage is an actual nodule. The second stage is referred to as the classification stage. This network takes predictions from the first-stage network and confirms or rejects the nodule predictions.

Out of the total dataset provided by our institute, the scans were divided into the training (176 scans), validation (44 scans), and test (491 scans) cohorts. The final evaluation was performed on the test cohort while we used the training and validation cohorts for fine-tuning our algorithm.

### 2.4. Datasets

In general, deep learning models require a large amount of data for them to learn and generalize to the task. Public datasets play a huge role in the development of deep learning algorithms, and, hence, we collaborated with a tertiary cancer care center to form a private dataset. Available datasets were used to train the models at different stages of the AI pipeline.

The data used in this study were taken from two sources. A total of 1599 patients were selected for the study. Out of these, 711(44.7%) patients were taken from the tertiary cancer care center and the remaining 888 (55.3%) patients were part of the LIDC-IDRI dataset. The Lung Image Database Consortium image collection (LIDC-IDRI) consists of diagnostic and lung cancer screening thoracic computed tomography (CT) scans with marked-up annotated pulmonary nodules [18]. Table 1 shows the distribution of cases among the different cohorts.

The 491 test cases were solely part of the 711 cases from the tertiary cancer care center. We observed that the patients provided by our hospital had a higher number of pulmonary nodules per patient as compared to the LIDC-IDRI dataset. The pulmonary nodule distribution is given in Table 2. The X axis is divided into three sections: data received from the institution, data from LUNA16 which is an open-source dataset, and total cases. Each section has two columns: the number of nodules present in the dataset and the number of unique patients with nodules considered in the study. The Y axis shows that during model training, the data are divided mainly into three sections. The column denotes the sample sizes used in the training, validation and testing phases of the model development. The nodule diameter in this study ranges from 3 mm to 30 mm. The annotations provided by radiologists and the LIDC-IDRI competition were in the form of a CSV containing x, y, and z center coordinates of the nodule along with the diameter of a nodule in mm. We decided to train a segmentation network to detect the nodules following participation in the Lung Nodule Analysis (LUNA16) challenge [19]. We converted the annotations into a segmentation mask by drawing a 3D sphere with the provided diameter and center coordinates. The pulmonary nodules in the training and validation cohorts of the hospital dataset were annotated by a team of radiologists with over six years of experience. The annotations were conducted using medical-grade BARCO monitors, which were routinely calibrated to ensure optimal display quality. All cases were independently reviewed and annotated by the team; however, granular details regarding the number of annotations performed by an individual were not available. The team members were blinded to the information from the other imaging modalities to minimize bias. Annotations were made by providing them with a tool to draw bounding boxes around the center slice of the nodule. Later, using the bounding box as a reference, we extracted similar information to the publicly available dataset to obtain the segmentation mask. Similarly, the test cohort was annotated by a different set of radiologists from our institute which was then used for the evaluation of the model.

### 2.5. Stages of AI Pipeline

**Figure 1:**
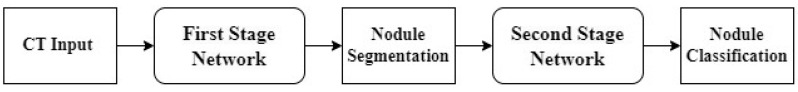
Overview of the two-stage network pipeline.

First-Stage Network: The first-stage network was trained to detect and segment the pulmonary nodules present in the dataset. We used a deep convolutional neural network (DCNN) architecture known as U-Net. The network performed downsampling until it reached a 4 × 4 × 4 shape, resulting in 4 group blocks in the downsampling path and 4 blocks in upsampling path. Each block had a residual connection making the network called the Residual U-Net [20] as shown in Figure 1. Each group block consisted of 2 convolutional layers with Batch Norm. We used the Relu activation function. Downsampling was performed with Maxpooling for each group of blocks with a 2 × 2 × 2 kernel. Upsampling was performed with the help of nn. Upsampling was performed with a scale factor of 2. The network took a 3D image as an input and then output the pixel-wise segmentation mask of the same size. The segmentation network was chosen to exploit the ability to segment each voxel of the nodule, providing the precise shape, centroid, and volume of the nodule. This information provides a wide range of characteristics that could help to better diagnose the nodules. We used dice loss to train the first stage. Since we did not use an object detection network to predict the bounding box coordinates, the term “detection” used here is in reference to the ability to determine the location of each nodule based on the blobs predicted by the segmentation network.

Second-Stage Network: We trained the second-stage network as a classification network to reduce the false-positive nodule predictions coming from the first stage. The network takes the nodule predicted by the first stage as the input and predicts whether to classify the input nodule as a true positive or false positive. Classifying the nodule as a true positive will keep the prediction made by the first stage in the final output; otherwise, it will reject it. The network architecture is similar to that of the first stage which predicts two outputs, which are a segmentation mask for the input and a classification of the input.

The network was trained to optimize the average of dice loss for training the segmentation output and cross-entropy loss for the classification output. As this was a classification task, more weightage was given to classification loss.

### 2.6. Training

**Figure 2:**
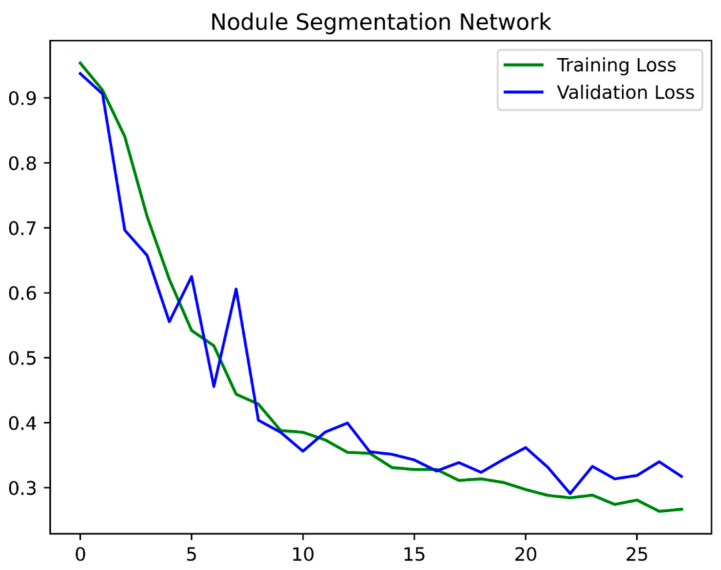
Training and validation curves for the first stage.

Convolutional neural networks cannot interpret voxel spacings natively, which is why we preprocessed our dataset by resampling all cases to a common median voxel spacing of [0.703125 × 0.703125 × 1.25] [20,21,22]. As the dataset consisted of a wide range of voxel spacing, we resized the training data into median spacing in order to provide more standardized input data for network training, which leads to better generalization. Typically, while training the networks, constraints like GPU memory might limit the size of the input that can be processed by a deep convolutional neural network. Hence, for the first stage, we trained the network with a 128 × 128 ×128 dimension which, in our experience, was able to retain enough spatial information to detect the nodule while optimally using the resources. We trained the first-stage network for 30 epochs starting with a 1e-3 learning rate and a batch size of 4 using the Adam optimizer. Figure 2 indicates the training and validation curves for stage 1. Training the network took 4 h per epoch on Nvidia GeForce RTX 2080Ti GPU.

**Figure 3:**
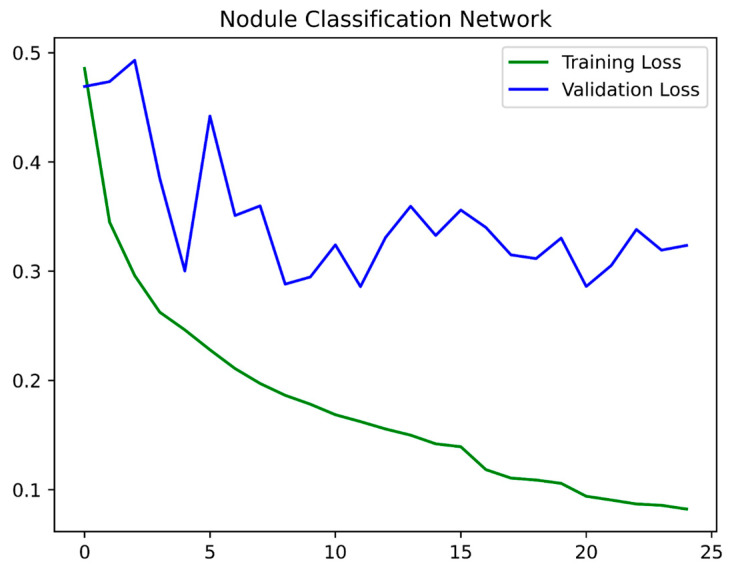
Training and validation curves for the second stage.

We used the same preprocessing methods for training the second-stage network. The input to the network was taken by cropping the area containing the detected nodule out of the CT scan and resizing it to the input size of 36 × 36 × 20. The network, similar to the first stage, was trained with Adam optimizer and a learning rate of 1 × 10^−3^. The training took 15 min per epoch and used 25 epochs. The training and validation curves are shown in Figure 3. The validation loss for the second-stage training does not appear stable, which could be the result of the smaller number of total nodules included in the validation, leading to each nodule gaining a higher weight during the loss calculation, hence creating unstable loss.

## 3. Results

We evaluated our deep learning model with 491 chest computed tomography (CT) scans provided by our hospital. Out of these, 401 scans contained 5669 nodules and the remaining 90 scans did not show any nodule.

### 3.1. Per-Lesion Performance

**Figure 4:**
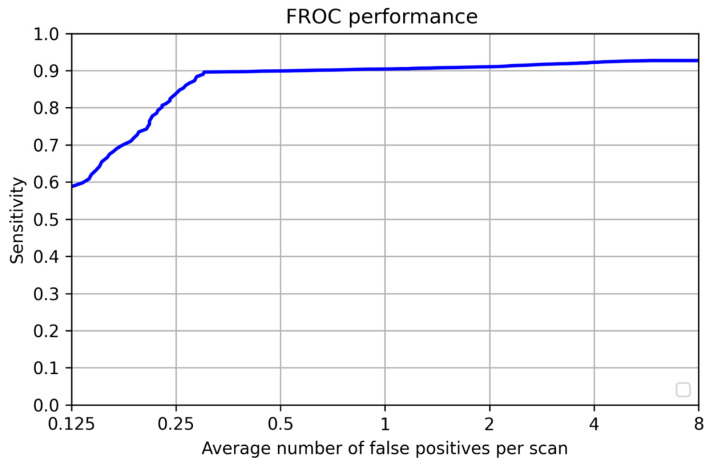
Test FROC curve.

The sensitivity for pulmonary nodule detections predicted by the 3D U-Net model was calculated using the annotations provided by an experienced radiologist. We also used free-response receiver operating characteristic (FROC) analysis. In the FROC curve, sensitivity was plotted as a function of the average number of false positives per scan. Figure 4 shows the FROC curve of our results. We used the FROC curve to decide on the threshold for the classification network which optimizes between the sensitivity and the number of false positives per scan. Our network achieved 0.90 sensitivity at only 0.3 false positives per scan as shown in Table 3. The X axis, the column labeled “Per-lesion performance”, denotes the performance of the total number of nodules included in the study across multiple metrics. Similarly, “Patient-wise performance” denotes the performance of the total number of unique patients involved in the study across multiple metrics.

The Y axis consists of various metrics to gauge the performance of the model on nodules and patients, namely, true positive (TP), false negative (FN), false positive (FP), true negative (TN), sensitivity, and specificity.

We also analyzed the nodule results based on the number of nodules present in a patient’s scan to explore the network’s prediction on different parameters. Table 4 shows a comparison between the patients’ scans which contain a nodule count between 1 and 6 as well as a higher number of nodules. As Table 4 suggests, our method achieved higher sensitivity irrespective of the number of nodules present in a patient’s scan. Our model was not able to perform well with nodules less than 3 mm in size, which according to the LUNA16 challenge [19] was not considered relevant for lung cancer screening protocols.

An overall score was calculated by using the CPM value. The CPM value was defined as the average of the sensitivity at seven predefined false-positive rates: 1/8, 1/4, 1/2, 1, 2, 4, and 8 FPs/scan, as shown in Table 5. Our network had a CPM score of 0.85.

### 3.2. Patient-Wise Performance

We analyzed the model performance based on patients to give us a clearer idea about the performance for patients with no nodules. We considered a patient as a positive scan when it had at least one nodule present; otherwise, it was negative. Table 3 shows that our model performed well, missing only 13 patients out of 401 scans giving a sensitivity of 0.95. Our model was able to identify all the negative scans without producing any false positives, achieving a specificity of 1.0.

Out of those thirteen patients, where our network did not predict nodules, nine patients had one nodule, three patients had two nodules, and one patient had three nodules. We also calculated the performance for patients who had nodule counts higher than six. If we predicted a number greater than six nodules in any of the scans, we considered that scan as a true positive. If the predicted nodule count was lower than seven, then it was considered a false negative. On a total of two hundred forty-nine patients, our model was able to achieve a patient-wise 0.99 sensitivity with two hundred forty-seven patients as true positives and two as false negatives.

## 4. Discussion

Our study is based on a subset of the 3D convolutional neural network called the U-Net, in which, to our knowledge, we have used for the first time a large number of nodules from a public dataset as well as from a tertiary cancer care center, which provides a varied spectrum of lung nodules to evaluate the performance of the algorithm in a better way, nullifying the biases relating to nodule characteristics. Our proposed AI pipeline achieved 90% sensitivity at only 0.3 false positives per scan over 491 CT scans provided by our institute. Since we have used a novel dataset, and given the fact that most of the previously published studies used only the LIDC-IDRI dataset, an exact comparison with the other studies would not be accurate or possible due to the unmatched datasets. Nonetheless, our model performed better with a better sensitivity and a significantly lower number of false positives per scan as compared to most of the published studies (Appendix A). This successful application demonstrates the effectiveness of using the 3D CNN U-Net for lung nodule candidate detection. The performance of the proposed scheme is comparable with that of other methods in the literature and is better than many of those. Among the usage of private datasets, Guo et al. [23] and Liu et al. [24] used small datasets comprising 29 scans (34 true nodules) and 32 scans (33 solitary nodules), respectively, for the evaluation of their proposed systems while Murphy et al. [25] used a larger private dataset of 813 scans for the evaluation of their proposed system and achieved a sensitivity of 80% with 4.2 FPs/scan, in which they used local image features and the k-nearest-neighbor classification. Their system’s sensitivity was lower than ours, despite their enormous dataset. In actual settings with a greater range of nodule types encountered in clinical scans, it is expected that systems with fewer datasets may perform poorly. Qin et al. [26] used a similar 3D CNN model as the backbone for their region proposal network (RPN), namely the U-Net architecture. Their research had a sensitivity of 98.2 percent with just four FPs/scan; but we had a significantly lower number of false positives with a substantially higher number of nodules. Our study has found better sensitivity and accuracy even with a higher number of nodules per patient, which is a special advantage in non-screening settings, for example, in an oncology setup dealing with various spectra of lung nodules in both primary cancer and metastases. Apart from this, in the testing dataset, our model was shown to perform better in cases with no nodules with a specificity of 100% signifying its huge impact for patients with negative findings, reducing the burden of additional investigations. We also found out that when using the private dataset, the performance of the model increases more than when just training with the publicly available datasets, as indicated in Table 6 and Table 7.

### Retraining Without Public Dataset

**Figure 5:**
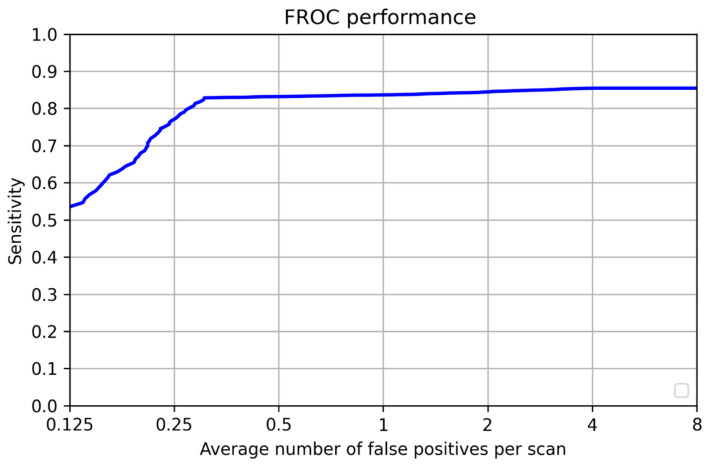
FROC curve for the experimental dataset which excluded the private cohort.

The network that was trained without the public dataset (just using data from our institute) achieved 0.83 sensitivity on the test data. For the same FPs/scan, training after adding the public dataset achieved 0.9 sensitivity. The CPM score also reduced with just training with the publicly available dataset, achieving a score of 0.787, while the network trained with the data provided by our institute achieved a score of 0.85. The highest sensitivity with eight false positives per scan was 0.857 without using a dataset from the tertiary cancer care center, shown in Figure 5. Our algorithm precisely provides the number of nodules present in the scan with the location in terms of the x, y, and z coordinates and the diameter of each nodule. This assists the radiologist in easily assessing the nodule and helps identify the potential risks.

**Figure 6:**
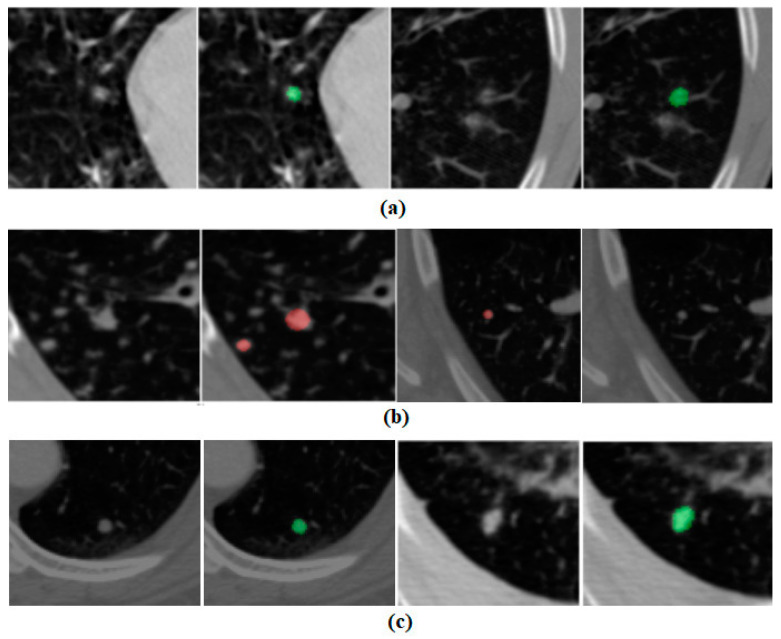
(**a**) False-positive examples, (**b**) false-negative example, and (**c**) true-positive examples.

In Figure 6, we present some cases of false positives, false negatives, and true positives predicted by our AI pipeline, respectively. This example also provides a peek into the reasoning of our deep learning model in predicting the nodule in a CT scan. The red markings shown in Figure 6 represent annotations by radiologists from our institute and the green markings show the segmentation mask provided by the AI pipeline. It is apparent that our AI pipeline is making mistakes in detecting nodules in some cases. Most of the false positives are coming from fibrotic bands, and veins in the chest CT scans which create an appearance like a nodule.

The main limitation of our study is that it is restricted to detection of the nodule; however, most of the recently performed studies focus on detection and characterization, which forms the future perspective of the model. Another limitation is that we have used a two-stage detector which is slower and can take up more memory than a single-stage detector; however, it comes with the tradeoff of having higher accuracy than single-stage detectors. This forms a future challenge for us: to achieve similar accuracy with a faster single-stage detector. To improve upon the current proposed study, additional contextual information about the nodules, such as linkages with nearby blood vessels, and information about the patient, such as the medical history report, can be added. In the next work, we will concentrate on making even more enhancements in order to better serve the medical community.

## 5. Conclusions

The findings show that our AI pipeline can easily aid the radiologist in better detection of pulmonary nodules and their characteristics in chest computed tomography (CT) scans. Automated accurate detection of these nodules will help in applications in mass screening programs, which will be beneficial in directing early diagnosis and the appropriate management of these nodules. The limitation of this study is its restriction to nodule detection, along with the other limitation of having used a two-stage detector which is slower and can take up more memory than a single-stage detector. These limitations form a future challenge for us: to expand beyond nodule detection and to achieve similar accuracy with a faster single-stage detector.

## Figures and Tables

**Table 1:** Breakdown of all the patients into training, validation, and test cohorts.

	OUR INSTITUTE	LUNA16	Total Cases
Training	176	710	886
Validation	44	178	222
Test	491	-	491
Total	711	888	1599

**Table 2:** The number of nodules amongst patients in different cohorts.

	OUR INSTITUTE		LUNA16		Total Cases	
	#Nodules	#Patients with Nodules	#Nodules	#Patients with Nodules	#Nodules	#Patient with Nodules
Training	743	144	929	484	1672	628
Validation	85	22	257	117	342	139
Test	5669	401	-	-	5669	401

**Table 3:** Per-lesion and patient-wise performance of the network on the test set.

	Per-Lesion Performance	Patient-Wise Performance
Total	5669	491
True Positive	5079	388
False Negative	590	13
False Positive	148	0
True Negative	-	90
FP per scan	0.3	-
Sensitivity	0.9	0.95
Specificity	-	1

**Table 4:** Summary of the results of the number of nodules per scan.

Nodule Count	Number of Patients	Sensitivity	FP	FN	TP
1–6	152	0.87	24	58	396
7–8	249	0.9	124	532	4683
9–10	227	0.88	44	126	932
11–19	87	0.88	68	147	1040
20–29	48	0.89	13	128	1020
30–39	8	0.92	2	22	261
40–49	9	0.93	7	29	383
50–59	7	0.89	8	43	338
60–59	4	0.94	4	16	246
70–79	3	0.88	2	26	192
80–89	6	0.95	0	24	480
90–99	1	0.85	0	16	90
≥100	1	0.88	0	13	97

**Table 5:** False positives per scan vs. sensitivity on the test and validation sets.

-	0.125	0.25	0.5	1	2	4	8	Mean
**Test**	0.58	0.84	0.9	0.9	0.91	0.92	0.92	0.85
**Validation**	0.63	0.72	0.83	0.86	0.87	0.87	0.87	0.807

**Table 6:** Per-lesion performance of the network which was trained without the inclusion of the public dataset.

Total True Nodules	5669
True Positive	4696
False Negative	973
False Positive	150
Sensitivity	0.828
FP per scan	0.3

**Table 7:** False positives per scan vs. sensitivity on the test set.

-	0.125	0.25	0.5	1	2	4	8	Mean
**Test**	0.53	0.77	0.83	0.836	0.84	0.85	0.857	0.787

## Data Availability

The datasets generated in this study are available on request to Abhishek Mahajan (drabhishek.mahajan@yahoo.in).

## Appendix A

**Table A1:** Comparison among different CAD schemes.

FP per Scan	Sensitivity (%)	Dataset	No. of Nodules	Total Scans	Year	Source
0.3	90	LIDC, Private	7683	1599	2021	Our Study
4	93.6	LIDC	1186	888	2019	Fu et al. [27]
4	90.7	LIDC	1186	888	2017	Chen et al. [28]
4	88.9	LIDC	698	502	2017	Ma et al. [29]
4	87.9	LIDC	1186	888	2016	Arinda et al. [30]
3.1	85.2	LIDC	631	98	2015	Lu et al. [31]
6.8	97.5	LIDC	148	84	2014	Choi et al. [32]
2	75	LIDC	68	108	2014	Brown et al. [33]
4.2	80	LIDC	103	84	2013	Teramoto et al. [34]
6.1	97	LIDC	148	84	2012	Cascio et al. [27]
4	87.5	LIDC	80	125	2011	Tan et al. [35]
4.2	80	Private	1518	813	2009	Murphy et al. [25]
5.6	76	LIDC, Private	241	85	2009	Sahiner et al. [36]
NA	94.7	Private	34	29	2009	Guo et al. [23]
4.6	93.7	Private	33	32	2009	Liu et l. [24]
0.4	848	Private	33	NA	2010	Sousa et al. [37]
4.8	80.3	Private	121	63	2003	Suzuki et al. [38]
8	80	LIDC	1749	949	2015	Torres et al. [39]
2	97	Private	NA	12	2012	Mabrouk et al. [40]
2.27	95.2	LIDC	151	58	2013	Choi et al. [32]
NA	95.3	LIDC	50	47	2016	Akram et al. [41]
3	95.7	LIDC	673	1010	2020	Wang et al. [42]
4	90.1	LIDC	1186	888	2016	Setio et al. [30]
10	80	SPIE-AAPM LUNG	NA	67	2016	Anirudh et al. [43]
4	94.4	LIDC	1186	888	2017	Ding et al. [44]
0.7	89.2	LIDC	1186	888	2018	Gruetzemac-her et al. [45]
2	95.2	LIDC	1166	888	2019	Kim et al. [46]
4	98.2	LIDC	1186	888	2019	Qin et al. [26]
